# The gut-liver axis in fatty liver disease: role played by natural products

**DOI:** 10.3389/fphar.2024.1365294

**Published:** 2024-04-15

**Authors:** Zhu Ming, Xie Ruishi, Xu Linyi, Yang Yonggang, Luo Haoming, Lan Xintian

**Affiliations:** ^1^ Changchun University of Chinese Medicine, Changchun, China; ^2^ School of Pharmacy, Changchun University of Chinese Medicine, Changchun, China; ^3^ Baicheng Medical College, Jilin, China

**Keywords:** gut-liver axis, NAFLD, ALD, natural products, plant metabolites

## Abstract

Fatty liver disease, a condition characterized by fatty degeneration of the liver, mainly classified as non-alcoholic fatty liver disease (NAFLD) and alcoholic liver disease (ALD), has become a leading cause of cirrhosis, liver cancer and death. The gut-liver axis is the bidirectional relationship between the gut and its microbiota and its liver. The liver can communicate with the gut through the bile ducts, while the portal vein transports the products of the gut flora to the liver. The intestinal flora and its metabolites directly and indirectly regulate hepatic gene expression, leading to an imbalance in the gut-liver axis and thus contributing to the development of liver disease. Utilizing natural products for the prevention and treatment of various metabolic diseases is a prevalent practice, and it is anticipated to represent the forthcoming trend in the development of drugs for combating NAFLD/ALD. This paper discusses the mechanism of the enterohepatic axis in fatty liver, summarizes the important role of plant metabolites in natural products in fatty liver treatment by regulating the enterohepatic axis, and provides a theoretical basis for the subsequent development of new drugs and clinical research.

## 1 Introduction

**FIGURE 1:**
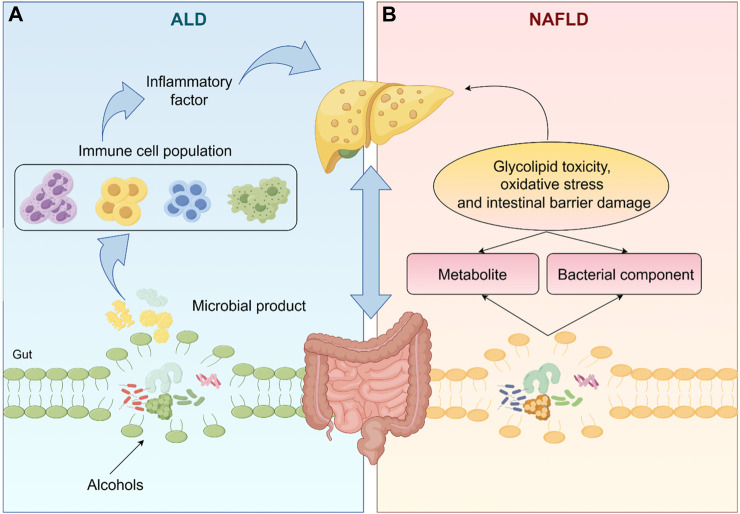
The gut microbiota and its metabolites directly or indirectly regulate liver gene expression, which ultimately leads to an imbalance in the gut-liver axis, leading to the occurrence of NAFLD and ALD. (The one-way arrows in the figure represent recursive relationships and the two-way arrows represent reciprocal relationships).

Fatty liver disease, a disease characterized by fatty degeneration of the liver, is mainly divided into NAFLD and ALD (Staufer and Stauber, 2023), has become a major cause of liver cirrhosis, liver cancer and mortality (Ko et al., 2023). The complex interactions between environment (especially diet), host genetics and gut microbiota are crucial for the development and progression of fatty liver disease (Wree et al., 2013). The gut liver axis is the bidirectional relationship between the gut, gut microbiota and liver (Albillos et al., 2020). The liver allows the bile ducts to communicate with the intestines and the portal vein transports the products of the intestinal flora to the liver (Tripathi et al., 2018). Intestinal flora and its metabolites directly and indirectly regulate hepatic gene expression, ultimately leading to an imbalance in the gut-liver axis, which contributes to the development of liver disease (Bauer et al., 2022), as shown in Figure 1.

In ALD, excessive alcohol consumption disrupts the intestinal barrier causing elevated levels of bacterial endotoxin in the portal circulation and dysbiosis of the intestinal flora thereby exacerbating inflammation levels and liver fibrosis (Szabo, 2015). There is now a large body of literature suggesting that targeting gut-liver axis homeostasis can be effective in improving ALD. For instance, dietary supplementation with propionate attenuates intestinal epithelial barrier dysfunction and prevents alcohol-induced liver injury (Xu et al., 2022). Probiotics can reverse alcohol-induced microbiota changes and prevent ALD progression by restoring gut microbial composition (Fuenzalida et al., 2021). Similarly, intestinal flora can use their metabolites to induce glycolipotoxicity, oxidative stress and intestinal barrier damage, while bacterial components such as Lipopolysaccharides (LPS), peptidoglycan, bacterial DNA and extracellular vesicles can be translocated to the liver via the damaged intestinal barrier, triggering immune cell hyperactivation and exacerbating the NAFLD response (Zhang et al., 2021; Pezzino et al., 2022).

In summary, regulating the homeostasis of the liver-gut axis is expected to provide a potential means to delay the progression of NAFLD. It has been reported that Gut Akkermansia muciniphila ameliorates can ameliorate NAFLD by modulating L-aspartate metabolism in the enterohepatic axis (Rao et al., 2021). Toll-like receptors (TLR) of intestinal flora modulate TLR ligands to stimulate liver cells to produce pro-inflammatory cytokines, which in turn affects the development of NAFLD (Miura and Ohnishi, 2014). Moreover, bile acid activated nuclear receptor and G protein-coupled receptors can drive NAFLD/Non-alcoholic steatohepatitis (NASH) disease progression (Xue et al., 2021). It follows that pharmacological modulation of the microbiota based on the gut-liver axis is a promising and useful therapeutic approach for fatty liver disease (Song and Zhang, 2022).

An increasing number of studies have confirmed that plant metabolites from natural medicines are an indispensable source for the development of hepatoprotective drugs. Although there is no large-scale clinical data as strong evidence to support the therapeutic efficacy of natural products, investigations have shown that phytomedicines play an important role in a wide range of diseases due to their broader biological activity, lower side effects and more diverse active ingredients (Yan et al., 2020). More and more pharmaceutical companies are now extracting medicinal plants to discover more potent natural medicines and their derivatives and to develop new drugs (He et al., 2022). At present, many natural products have been found to have the effect of regulating the intestinal liver axis and improving fatty liver. These natural products are mainly effective by affecting intestinal microbiota, bile acid metabolism, inflammatory reactions and other ways (Cao et al., 2023). This article presents a comprehensive review of how metabolites derived from natural products influence the gut microbiota composition. We summarize the research progress of plant metabolites in improving fatty liver disease through the “gut-liver axis” and discuss their potential development trends and shortcomings.

## 2 Fatty liver disease and gut-liver axis

### 2.1 ALD and the gut liver axis

ALD is a liver damaging disease caused by excessive and chronic alcohol intake, including fatty liver, alcoholic steatohepatitis (ASH), alcoholic hepatitis (AH), cirrhosis, and hepatocellular carcinoma (HCC) (Yang et al., 2022). Excessive alcohol consumption can cause cell damage, inflammation, oxidative stress (Louvet and Mathurin, 2015) and can also disrupt the intestinal barrier (Park et al., 2016). After the intestinal barrier is disrupted, the composition of the gut microbiome changes due to alcohol consumption (Bull-Otterson et al., 2013), which leads to an increase in the transport of microbial products such as LPS from the intestines to the liver via the portal circulation, resulting in the activation of immune cells and the production of high levels of the pro-inflammatory cytokines TNF-α, IL-1β and IL-6, which in turn contributes to the vicious cycle of ALD (Leclercq et al., 2014). In one study it was noted that The relative abundance of *Mycobacterium avium* and *Mycobacterium tuberculosis* bacteria in the gastrointestinal tract of mice fed alcohol for 3 weeks increased significantly (Yan et al., 2011). Moreover, we learned that intestinal permeability and LPS were significantly increased in alcohol-dependent subjects at the onset of withdrawal, suggesting that the gut-liver axis plays an important role in the pathogenesis of alcohol dependence (Leclercq et al., 2012). Gao et al. (2023) showed that oral administration of Porphyromonas gingivalis worsened hepatic inflammation in ALD mice by increasing the protein expression of Toll-like receptor 4 (TLR4) and p65, increasing the mRNA expression of IL-6 and TNF-α and up-regulating the production of Transforming Growth Factor β1 (TGF-β 1) and galactaglutinin-3 (Gal-3). Likewise, in alcoholic cirrhosis the intestinal flora remains altered and translocated toward the liver and ascites (Oesterreicher et al., 1995; Naseri et al., 2021). Currently, there is a plethora of treatments based on the gut-liver axis. In 2018, a study proposed that bile acid-FXR-FGF15 signaling could be improved by modulating hepatic Cyp7a1 and lipid metabolism to reduce ALD in mice (Hartmann et al., 2018). *Lactobacillus* plantarum KLDS1.0344 and *Lactobacillus* acidophilus KLDS1.0901 Mixture prevents ALD in mice by protecting the intestinal barrier (Hou et al., 2021). Patel’s Team Finds Visbiome Prevents Alcohol-Induced Cell Damage, ER Stress, Oxidative Stress and Regulates Lipid Metabolism (Patel et al., 2021; Sun et al., 2022). In conclusion, it is significant to further explore the enterohepatic axis of alcoholic liver disease.

### 2.2 NAFLD and the gut-liver axis

The gut-liver axis plays a key role in the pathogenesis of NAFLD and is associated with disease severity (Chen and Vitetta, 2020). In NAFLD, the abundance of Aspergillus spp. will increase. In cirrhosis, the Bacteria such as Prevotella and Verotella invade the distal gut (Aron-Wisnewsky et al., 2020a). In contrast, the abundance of *M. avium* was significantly increased in NASH patients with concomitant hepatic fibrosis and the abundance of B. Prevotella was decreased (Boursier et al., 2016). It is well known that obesity is one of the characteristics of NAFLD, but there are some patients who are thin and they are called lean nonalcoholic fatty liver disease (Seto and Yuen, 2017; Albhaisi et al., 2019). Almost all men with lean nonalcoholic fatty liver disease have been reported to lack the ability to produce estragole and they have reduced abundance of Slackia (Iino et al., 2022). In addition to changes in the composition of the gut microbiota, components from the gut microbiota (LPS, peptidoglycans, DNA, and extracellular vesicles) (Ji et al., 2019a) and metabolites (bile acids, short-chain fatty acids, amino acids, choline and ethanol) (Leung et al., 2016) have emerged as key factors regulating the pathologic process of NAFLD. Several studies have shown that gut microbiota disorders lead to reduced synthesis of secondary bile acids, which in turn reduces the activation of nuclear receptors such as farnesol X receptor (FXR), pregnane X receptor, Takeda G protein-coupled bile acid protein 5 and vitamin D receptor leading to NAFLD (Chen et al., 2019; Jayachandran and Qu, 2023). Moreover, the intestinal metabolite sodium butyrate can prevent the progression of NAFL to NASH by promoting hepatic glucagon-like peptide-1 recetor (GLP-1R) expression (Zhou et al., 2018). Tryptophan metabolism in NALFD has been shown to increase inflammation and lipogenesis and lower the intestinal barrier by decreasing its metabolite indole (Ji et al., 2019b). Ethanol, as the biggest causative agent of ALD, should not be ignored in NAFLD as well. As early as 2000, ethanol was recognized as a player in the pathogenesis of NAFLD (Cope et al., 2000). Yuan et al. (2019) demonstrated that *Klebsiella pneumoniae* K14, which produces large amounts of alcohol, is the cause of NAFLD. New therapeutic approaches to modulate the gut microbiota by administering probiotics, prebiotics, synbiotics and antibiotics have been proposed (Cho et al., 2018; Coskun and Celep, 2022), such as *Lactobacillus* plantarum MA2 to reduce hepatic cholesterol and triglyceride levels and to increase fecal *Lactobacillus* and Bifidobacterium populations (Wang et al., 2009). *Lactobacillus* rhamnosus PL60 produces large amounts of conjugated fatty acids that can effectively ameliorate NAFLD (Lee et al., 2006; Lee and Lee, 2009). Therefore, therapies that effectively target the gut microbiome may be beneficial in the treatment of patients with NAFLD (Jayakumar and Loomba, 2019).

## 3 Role of natural products and their extracts in fatty liver disease

Here, we describe plant-derived active ingredients that modulate the structure of disturbed intestinal flora under disease conditions, influence the metabolic processes of some specific flora and alter the production of intestinal flora metabolites to ameliorate fatty liver disease in a variety of cellular and animal models. These natural products are mainly isolated or extracted from plants and can be broadly categorized into alkaloids, saponins, phenols, polysaccharides, terpenoids and flavonoids (Table 1).

**TABLE 1:** Mechanism of natural products and their extracts in the treatment of fatty liver through the gut-liver axis.

Compound type	Compound name	Machine	Diseases	References
Alkaloid	Berberine	• Upregulation of intestinal gene expression of fasting-induced adipokine (Fiaf) in mice	NAFLD	Zhang et al. (2015)
		• Increases the ability of short-chain fatty acids (SCFAs) to produce bacteria		
		• Reduces intestinal endotoxin entering the bloodstream		
		• Reduces inflammation, insulin resistance		
		• Inhibition of the phylum Thick-walled Bacteria and Bacteroidetes		
	Berberine	• Promoting expansion of immunosuppressive cell populations	ALD	Li et al. (2020b)
		• Reduces slime molds		
Saponin	Ginsenoside Rh4	• Improvement of hepatic steatosis	NAFLD	Yang et al. (2023)
		• Improvement of lobular inflammation levels		
		• Increased levels of intestinal SCFAs and BAs		
	Ginsenoside Rk3	• Improved the composition of intestinal flora in mice	NAFLD	Guo et al. (2023a)
		• Decrease the abundance of harmful bacteria such as Phylum Thickettsiae		
		• Increase beneficial bacteria		
	Astragaloside	• Decrease the abundance of harmful bacteria	NAFLD	Zhou et al. (2021)
		• Increase the abundance of beneficial bacteria with anti-inflammatory effects (e.g., Clostridia, Lactobacillaceae, and Buttercupaceae)		
	Panax ginseng saponin	• Slow down the rate of SCFAs from the intestine to the liver	NAFLD	Xu et al. (2021)
		• Inhibit TLR4 to promote AMPKα activation to reduce lipogenesis in hepatocytes		
		• Alleviate intestinal leakage phenomenon (intestinal permeability) by enhancing the expression of the tight junction proteins Claudin-1 and ZO-1		
Phenol	Eugenol	• Increase the expression of GLP-1 receptor (GLP-1R) in the duodenum, liver, arcuate (ARC) and paraventricular nucleus (PVN) and c-fos in the nucleus tractus solitarius (NTS) by modulating the gut-brain-hepatic axis of glucagon-like peptide-1 (GLP-1)	NAFLD	Li et al. (2022a)
	Paeonol	• Decrease fungal abundance	NAFLD	Wu et al. (2020)
		• Block glucan translocation to the liver		
	Sorbiferin	• Improve abnormal lipid metabolism	NASH	Qu et al. (2022)
		• Regulates the composition of gut microbiota		
		• Reverses FXR deficiency		
	Baicalein	• Improved MCD diet-induced knot length	NAFLD	Guo et al. (2023b)
		• Restored mucosal barrier integrity by upregulating tight junction protein intestine in mice		
	Lignans	• Improve intestinal mucosal permeability to high-fat diet-induced increase in fluorescein isothiocyanate-dextran (FD4) by modulating the expression of tight junction proteins in the intestinal tract, restoring intestinal barrier function	NAFLD	Liu et al. (2021)
		• Decreasing relative abundance ratios of the phylum Thickwell/anaplasmid phylum		
Polysaccharide	Salvia miltiorrhiza polysaccharide	• Regulate the homeostasis of the intestinal microbiota	NAFLD	Li et al. (2022b)
		• Upregulate the expression of integral membrane proteins (Claudin and Occludin) and the junction complex protein ZO-1 in the jejunum and colon		
	Echinacea polysaccharide	• Increase the abundance of *Serratia marcescens*, *Lactobacillus*, and Synechococcus, and decreasing the abundance of *Escherichia coli* and *Enterococcus*	ALD	Jiang et al. (2022)
		• Increase production of n-butyric acid		
	Astragalus polysaccharides	• Improve the ratio of intestinal flora, and the abundance of harmful flora decreased significantly	ALD	Zhou et al. (2021)
	Comfrey polysaccharide	• Upregulate the expression of ileocecal tight junction proteins	NASH	Jiang et al. (2021)
		• Decrease the entry of enteric endotoxin into the portal circulation		
		• Attenuate hepatic inflammation and injury		
	Cordyceps Sinensis polysaccharide	• Reduce the number of enterococci in the cecum	NASH	Chen et al. (2020); Wu et al. (2022)
		• Regulate the metabolism of bile acids in the intestine		
		• Increase the proportion of actinomycetes		
		• Increase the degree of intestinal flora disruption		
	MDG-1	• Regulate the balance of the gut microbiota	NASH	Wang et al. (2019)
		• Increase the relative abundance of beneficial bacteria		
Glycosides	Cornelianoid glycoside	• Reverse alcohol-induced changes in the tight junction proteins ZO-1 and occludin	ALD	Han et al. (2021)
		• Reduce serum levels of LPS, and blocks the hepatic inflammatory response caused by LPS stimulation		
Terpenoids	Glycyrrhizic acid	• Modulate the relative abundance of Trichoderma, Trichoderma, *Helicobacter* and *Enterobacter*	NAFLD	Wang et al. (2022a)

### 3.1 Alkaloid

Alkaloids are a class of nitrogen-containing organic secondary metabolites with diverse structures and have a wide range of biological functions, including anti-inflammatory, antioxidant, antitumor and immunomodulatory effects (Liu et al., 2023; Omidkhoda et al., 2023). Several studies have shown that the effects of alkaloids on NAFLD/ALD are mediated through the regulation of gut flora.

Berberine (BBR), an isoquinoline alkaloid derived from *Coptis chinensis* Franch., has long been used clinically against intestinal bacterial infections and has been shown to have significant efficacy in the treatment of NAFLD. Research indicates that BBR exhibits low absorption into the bloodstream, with a significant accumulation in the intestines where it exerts its pharmacological effects locally. BBR affects the gut-liver axis at several interrelated levels, including modulation of the gut microbiota structure, alteration of gut microbe-derived metabolites, etc., which reduces microbial exposure and the pro-inflammatory environment of the liver and modulates liver metabolism (Peng et al., 2019; Dehau et al., 2023; Zhu and Li, 2023). Zhang et al. (2015) collected feces from C57 mice fed a high-fat diet (HFD) for 10 weeks and collected feces from non-alcoholic hepatitis model mice fed BBR (100 mg/kg body weight) for eight consecutive weeks and performed 16sRNA sequencing. Comparing the feces from mice fed a HFD at weeks 0, 10, and 18, they found that BBR significantly altered the relative abundance of the phylum Thick Walled Bacteria (THWB) and Bacteriodesium anisopliae (BAP) in a concentration-dependent manner (Zhang et al., 2015). At the onset of obesity, which is generally characterized by a decrease in the anabolic phylum or an increase in the thick-walled phylum, BBR participates in the regulation of hepatic energy metabolism by influencing fatty acid metabolism through modification of the composition of the intestinal flora and increasing the abundance of short-chain fatty acid-producing bacteria.

Piperine (PIP) found in large quantities in *Piper nigrum* L. that has anti-obesity, anti-inflammatory, and hepatoprotective properties (Cheng et al., 2023; Joshi et al., 2023). *In vitro* studies have found that PIP regulates lipid metabolism in HepG2 cells in a Bmal1/Clock-dependent manner (Zhang et al., 2022) and *in vivo* studies have found that PIP ameliorates insulin resistance in diabetic mice constructed by subcutaneous injection of monosodium glutamate (MSG) in neonatal mice (Liu et al., 2020). Piperine was administered at 20 and 40 mg/kg body weight per day in an obese mouse model induced by high-fat dietary feeding. It was found that piperine at high doses significantly reduced body weight, liver weight, perirenal fat weight and serum triglyceride, total cholesterol, LDL-cholesterol and glucose levels. In addition, piperine significantly attenuated the expression of SREBP-1c mRNA and downregulated the expression of IL-6. 16S rRNA sequencing showed that piperine increased the diversity of gut microbiota and the relative abundance of Muribaculaceae and Ruminococcaceae was significantly increased, while Dubosiella and Enterorhabdus were inhibited by piperine. were inhibited by piperine (He et al., 2022). IL-6 expression is closely associated with macrophage M1 polarization in adipose tissue and PIP may play an important role in fatty liver disease by improving the structure of the intestinal flora and thereby affecting immune cell homeostasis. Another study showed that PIP also demonstrated its ability to downregulate jejunal tumor necrosis factor-α, reduce lipopolysaccharide-induced proliferative damage of intestinal cells, enhance the intestinal barrier function and inhibit intestinal fatty acid absorption in cellular and animal models in in vivo and *in vitro* studies in Caco-2 cells and HFD-fed C57 mice for 16 weeks (Wang et al., 2021).

Capsaicin (CAP), a metabolite of *Capsicum frutescens* L., it has a long hydrophobic carbon end with a polar amide group and a benzene ring (Karimi-Sales et al., 2024), plays a role in the metabolic processes of energy homeostasis and fatty acid oxidation (Saito and Yoneshiro, 2013), making it a focus of research in obesity treatment. Clinical studies have shown that moderate amounts of capsaicin can promote metabolism and improve blood glucose regulation, as well as increase satiety and reduce overeating. To further explore its mechanism of action, it was studied *in vivo* in mice. Hui et al. (2020) state that CAP specifically activated hepatic transient receptor potentialvanilloid 1 cation channels in HFD-induced C57BL/6J NAFLD mice, increased Ca^2+^ in-flux, promoted hepatic fatty acid β-oxidation and decreased hepatic TG content and lipid deposition. CAP has limited its development due to its poor water solubility low bioavailability and its gastrointestinal mucosal irritation effect. Currently, capsaicin is gradually being used as a prodrug in nanoparticles for administration to increase bioavailability, reduce gastric mucosal irritation and improve its therapeutic potential for NAFLD and ALD (Feng et al., 2018).

Koumine (KM), derived from the stem and leaves of *Gelsemium elegans* (Gardner & Champ.) Benth., has significant anti-inflammatory effects antitumor, antioxidant, immunomodulatory and hepatoprotective effects (Wang et al., 2022; Que et al., 2023). Its immunomodulatory effect on rats with NAFLD has been demonstrated in recent years. Koumine was intraperitoneally injected into SD rats fed with cholesterol 1%, bile salt 0.1%, lard 10%, egg yolk powder and whole milk powder 5% and pro-inflammatory factors in their serum such as cytokine interferon-c, IL-17A, MCP1 and IL-1β were significantly suppressed, which was associated with its inhibition of Th17 cell differentiation and increase in the number of Treg cells. IL-17A, TNF-a, IL-6, MCP1 and IL-1β were significantly inhibited, which was closely related to the inhibition of Th17 cell differentiation and the increase in the number of Treg cells (Yue et al., 2019). A study using concanavalin A (Con A)-induced autoimmune hepatitis (AIH) model in mice verified the pharma-cological activity of KM in steatohepatopathy, KM activated the Nrf2 pathway, upregulated the expression of antioxidant factors HO-1 and Nrf2 and downregulated the expression of Keap1. In addition, the NF-κB signaling pathway was inhibited. Also KM significantly improved the composition of the gut microbiota and increased the abundance of beneficial bacteria (Que et al., 2023).

### 3.2 Saponin

Saponins, consisting of saponin elements as their glycosidic elements and carbohydrate chains, are widely found in plants and are prevalent in *Astragalus mongholicus* Bunge as well as *Panax ginseng* C.A.Mey., exerting their medicinal effects mainly through interactions with the gastrointestinal environment and intestinal microbiota (Zhang et al., 2023).

Ginsenoside is the main natural product of ginseng and has a variety of pharmacological activities. Ginsenoside Rh4 is obtained by eliminating the water molecule at the C20 position of Rh1. Extensive pharmacological studies have shown that ginsenoside Rh4 can be used as an anticancer, anti-inflammatory and anti-oxidative stress agent. Recent studies have demonstrated that ginsenoside Rh4 can improve the balance of intestinal flora. In the NAFLD model mice constructed on a Western diet and CCL4, Ginsenoside Rh4 significantly increased the levels of intestinal SCFAs and bile acids (BAs) while ameliorating hepatic steatosis and lobular i-nflammation levels (Yang et al., 2023), all of which were attributed to its alteration ofthe gut microbiota composition.

Studies have shown that ginsenoside Rk3 can intervene in high-fat diet-induced repair of intestinal barrier dysfunction in C57BL/6 mice, increase the expression of tight junction proteins and reduce the level of inflammatory cytokines, inhibit the TLR4/NF-B signaling pathway, si-gnificantly reduce the ratio of Firmicute/Bacteroidete, which effectively improves the metabolic dysfunction of the intestinal flora and inhibits the inflammatory cascade response (Chen et al., 2021). Another study showed that Rk3 ameliorates dimethylnitrosamine- and CCl4-induced intestinal dysbiosis in a mouse model of HCC, resulting in the inhibition of the LPS-TLR4 signaling pathway, which plays a key role in the prevention of HCC (Qu et al., 2021). Guo et al. (2023) showed that ginsenoside Rk3 was effective in reducing the number of harmful bacteria and increasing the number of beneficial bacteria in the feces of NAFLD mice fed a high-fat, high-cholesterol diet and injected with CCl4, thereby improving the composition of the intestinal flora of the mice.

As a legume plant, *Astragalus mongholicus* Bunge, one of the plant metabolites, has been applied commonly in China due to its biological activities, such as antioxidant, anti-inflammatory, hepatoprotective, immunomodulating, anti-cancer, and anti-photoaging properties. Triterpene saponins and polysaccharides are believed to be the two main natural products in Astragalus. *In vivo* studies have confirmed that astragalus saponin improves the gut microbial structure of alcohol-induced ICR mice. The gut flora of alcohol-induced ICR mice showed an increase in the number of harmful bacteria (Gardnerella and unclassified_p_thickness) and a decrease in the number of beneficial bacteria (Ackermansia). After treatment with high doses (300 and 600 mg/kg bw) of astragaloside, the ratio of intestinal flora in ALD mice significantly improved and the number of harmful bacteria decreased. In addition, the number of *Lactobacillus*, a beneficial bacterium that regulates inflammation, was significantly increased (Zhou et al., 2021). From this we can see that the synthesis of drugs using astragalus saponin as the main ingredient is very promising and may contribute to the future development of new drugs for metabolic lipid disorders. However, there is still no clinical efficacy information available and more research is needed to assess the safety and efficacy of its application, clinical trials with a large increase in patient populations should be conducted.

Xu et al. (2021) showed that Panax notoginseng saponin (PNS) from *Panax notoginseng* (Burkill) F.H.Chen could slow down the rate of SCFAs from the intestine to the liver and inhibit TLR4 to promote AMPKα activation to reduce lipogenesis in hepatocytes and alleviate intestinal leakage phenomenon (intestinal permeability) by enhancing the expression of the tight junction proteins Claudin-1 and ZO-1, which are important for the intestinal-hepatic axis, through These three sets of pathways exert anti-adipogenic and anti-fibrotic effects. This finding was mainly obtained from *in vivo* studies with high-fat diet (HFD)-induced obese mice and obesity-prone Lep^ob^ mice. PNS exerts pharmacological activity in NAFLD/ALD by improving the gut microbiota through multiple pathways, what are the key mechanisms should be further explored.

### 3.3 Phenol

Phenols are mainly produced by plants through various metabolic pathways including phenols/phenolic acids, flavonoids, stilbenes and lignans (Deka et al., 2022).

Eugenol, a natural products are sourced from many aromatic botanic drugs, *in vivo* experiments were conducted using Wistar rats (200 g ± 20 g) to construct a NAFLD model, after gavage of eugenol, the liver index of rats decreased in a dose-dependent manner and the study of the mechanism revealed that eugenol could increase the expression of GLP-1R in the duodenum, liver, arcuate (ARC) and paraventricular nucleus (PVN) and c-fos in the nucleus tractus solitarius (NTS) by modulating the gut-liver axis of glucagon-like peptide-1 (GLP-1), providing a novel strategy for the treatment of NAFLD (Li et al., 2022).

Paeonol, which also has a wide range of biological activities, is derived from the dried bark of *Paeonia lactiflora* Pall., which belongs to Paeoniaceae (Ranunculaceae) or the dried root of Xu Chang qing (Asclepiadaceae) or the whole grass (Li and Gu, 2022). Hu et al. (2010) reported experiments on the treatment of alcoholic liver injury rats with paeonol, which resulted in paeonol decreasing the level of ALT, the level of reduced hepatic gene expression of lipogenic genes (*p* < 0.05) without affecting hepatic CYP2E1 protein expression, significantly reduced serum and tissue levels of inflammatory cytokines, tissue lipid peroxidation, neutrophil infiltration and inhibited hepatocyte apoptosis (*p* < 0.05), thereby reducing hepatocyte injury. It has also been reported to ameliorate acute alcoholic hepatitis-associated liver injury by decreasing fungal abundance and blocking glucan translocation to the liver (Wu et al., 2020). A study of a C57BL/6 mouse model of *Candida* albicans-induced ALD treated with 480 mg/kg of Paeonol found that the mechanism of action was mainly through the Dectin-1/TLR2/NLRP3 pathway (Xiao et al., 2023).

Sorbiferin (SDS) in *Rhodiola rosea* L. (Hu et al., 2021), improves abnormal lipid metabolism, regulates the composition of gut microbiota (Qu et al., 2022), effectively reverses FXR deficiency during NASH. SDS was intragastrically administered at a dose of 20 mg/kg/day for 4 weeks. After treatment with salidroside, liver steatosis, TG content and serum inflammatory factors significantly improved and HFD-induced intestinal bacteria, bile acid disorder and FXR deficiency were significantly alleviated (Li et al., 2020). The mechanism of action of SDS is still obscure, but some studies have demonstrated that SDS attenuates HFD-induced NAFLD by inhibiting fatty acid uptake-associated (Cd36 and Fabp1) and synthesis-associated (Fasn, Pparg, Scd1 and Srebf1) factors (Hu et al., 2021).

The isolated metabolite Luteolin from *Reseda odorata* L. exerts pharmacological activity in repairing the intestinal mucosal barrier and microbiota imbalance in NAFLD rats. Luteolin have been reported to improve intestinal mucosal permeability to high-fat diet-induced increase in fluorescein isothiocyanate-dextran (FD4) by modulating the expression of tight junction proteins in the intestinal tract, restoring intestinal barrier function, improving NAFLD by decreasing relative abundance ratios of the phylum Thickwell/anaplasmid phylum. In this study, 48 Wistar rats (6 weeks old) were used and all the rats were randomly divided into 6 groups (*n* = 8): normal diet group (ND), high fat diet group (HFD), low dose lignocaine group (L5mg/kg/day), medium dose lignocaine group (50 mg/kg/day) and high dose group (100 mg/kg/day). The therapeutic effect of lignocaine on hepatic inflammation was dose dependent (Liu et al., 2021).

In contrast, flavonoids, as important phenolic compounds, are widely distributed in a wide range of plant products and enter the circulatory system mainly through the intestines to exert their beneficial effects (Li et al., 2023). Baicalein, a dietary flavonoid extracted from *Scutellaria baicalensis* Georgi, can improve lipid levels and hepatic steatosis in choline-deficient diet-induced NAFLD mice through a multi-target and multi-channel mechanism (Zhang et al., 2022). Guo et al. (2023) demonstrated that baicalein significantly improved Methionine-Choline Deficient (MCD) diet-induced knot length and restored mucosal barrier integrity by upregulating tight junction protein intestine in mice. In this study, mainly male C57BL/6 mice were used, administered by gavage at 200 mg/kg and 400 mg/kg, respectively, with higher doses showing better therapeutic effects.

### 3.4 Polysaccharide

Polysaccharides are the main metabolites of botanical drugs, their biosynthesis is partly controlled by genes as well as by various environmental factors. In recent decades, polysaccharides isolated from different kinds of botanical drugs have received widespread attention for their important biological activities such as antitumor, antioxidant, antidiabetic, radiation, antiviral, hypolipidemic and immune regulation (Zeng et al., 2019). Summarizing the existing reports we can easily find that the polysaccharide components of several botanical drugs play an important role in fatty liver disease.

*Salvia miltiorrhiza* Bunge is widely used in the treatment of cardiovascular diseases and liver injury (Fu et al., 2023). The addition of Salvia miltiorrhiza effectively attenuates MCD diet-induced hepatic steatosis and inflammation in C57BL/6 mice, mainly due to its ability to alter the structure of the intestinal microbiota and partially reverse intestinal ecological dysregulation (Li et al., 2021). The polysaccharide component of Salvia miltiorrhiza can effectively regulate the homeostasis of the intestinal microbiota. It has been shown to upregulate the expression of integral membrane proteins (Claudin and Occludin) and the junction complex protein ZO-1 in the jejunum and colon (Li et al., 2022). It has also been demonstrated that danshen polysaccharides modulate the relative abundance in the intestinal flora and ameliorate HFD-induced intestinal dysbiosis (Li et al., 2022).

Echinacea polysaccharide (EPP), a homogeneous polysaccharide, was studied microbiomically and metabolomically in alcoholic model mice and found to reverse alcohol-induced gut microbiota disruption by increasing the abundance of *Serratia marcescens*, *Lactobacillus* and Synechococcus, and decreasing the abundance of *Escherichia coli* and *Enterococcus*. Also, EPP promotes increased production of n-butyric acid, a short-chain fatty acid that maintains the integrity of the intestinal barrier (Jiang et al., 2022).

Some *in vivo* studies in which we found significant effects of polysaccharide natural products. High-dose treatment of astragalus polysaccharides in ALD mice significantly improved the ratio of intestinal flora, and the abundance of harmful flora decreased significantly (Zhou et al., 2021). Jiang et al. (2021) used ethanol stepwise precipitation to obtain three comfrey polysaccharide fractions (EPP40/60/80), of which EPP40 had the most pronounced effect in up-regulating the expression of ileocecal tight junction proteins, decreasing the entry of enteric endotoxin into the portal circulation, and attenuating hepatic inflammation and injury. *Cordyceps sinensis* (BerK.) Sacc. is a common botanical drugs with a variety of pharmacological activities including repair, antioxidant and inhibition of apoptosis (Gu et al., 2015). Cordyceps sinensis can reduce the number of enterococci in the cecum and regulate the metabolism of bile acids in the intestine (Wu et al., 2022). Further studies on Cordyceps Sinensis polysaccharide (CSP) revealed that CSP increased the proportion of actinomycetes, increased the degree of intestinal flora disruption, led to further development of NASH (Chen et al., 2020). MDG-1, a β-D-fructan extracted from the root of *Ophiopogon japonicus* (Thunb.) Ker Gawl. MDG-1 regulates the balance of the gut microbiota and increases the relative abundance of beneficial bacteria during the onset of NASH. When MDG-1 is degraded and utilized by the intestinal microbiota, it can increase the content of acetic acid and valine, which affects hepatic lipid accumulation (Wang et al., 2019). The role played by glycosides in hepatoprotection has been previously demonstrated. For example, cornelianoid glycoside (CIG), a glycoside extracted from the fruit of *Cornus officinalis* Siebold & Zucc., has a more prominent role in ameliorating alcohol-induced intestinal damage. The mechanism is mainly that CIG reverses alcohol-induced changes in the tight junction proteins ZO-1 and occludin, reduces serum levels of LPS and blocks the hepatic inflammatory response caused by LPS stimulation (Han et al., 2021).

Polysaccharides are essential biomolecules in organismal activities, usually consisting of 10 or more monosaccharides linked by different types of glycosidic bonds, are widely found in botanical medicines. With the deepening of research, researchers have confirmed the biological activities of polysaccharides in regulating lipid metabolism disorders, regulating intestinal flora and oxidative stress (Hu et al., 2020). Since most of the polysaccharides are mixtures, there are few reports on the exact molecular structure, which also limits the conformational relationship of polysaccharides in the prevention and treatment of metabolic fatty liver disease. It is necessary to carry out in-depth studies on the pharmacological targets of polysaccharides in improving NAFLD or ALD on the basis of the existing studies, so as to provide theoretical support for the development of polysaccharides wOther plant metabolite.

### 3.5 Terpenoids

*Glycyrrhiza aspera* Pall. is a perennial botanical drug, several of its metabolites play an important role in NAFLD (Sun et al., 2017), such as glycyrrhizic acid, glycyrrhizic flavonoids, etc. The metabolite that has been reported to treat NAFLD via the enterohepatic axis is glycyrrhizic acid. The researchers performed modelling by randomly dividing 40 male Sprague-Dawley rats (6 weeks of age) into 5 groups fed a high-fat diet (45% energy from fat; 4.73 kcal/g; D18040307; SYSE Co., Ltd., Changzhou, China), administering 40 mg/kg glycyrrhetinic acid daily to the groups. The group was administered 40 mg/kg glycyrrhetinic acid per day. The body weight of each rat was measured every 3 weeks. At the end of the 12th week, the faeces of the rats were collected and sequenced by 16sRNA, which showed that the glycyrrhizic acid group significantly increased the relative abundance of Peptostreptolysin and decreased the relative abundance of Lachnospiraceae and Coriobacteriaceae (Wang et al., 2022). However, the mechanisms by which such metabolites improve intestinal flora have not been well studied.

Plant-derived substances have a number of advantages over animal, mineral and microbial sources, such as the fact that they are often renewable, which makes them more sustainable than animal and mineral sources, can be grown and regenerated through natural processes; they are more readily accepted and absorbed by living organisms due to a high degree of similarity between the environment they require and the environment in the body, they are relatively inexpensive to grow and collect, as compared to animal-derived substances (e.g., animal proteins) and mineral sources (e.g., metallic minerals). At the same time, compared with animal-derived substances (such as animal protein) and mineral resources (such as metal minerals), plant-derived substances are relatively inexpensive to grow and collect. Therefore, plant metabolites in natural products have been widely used in regulating the enterohepatic axis to improve NAFLD/ALD. However, we summarise the current research and find that the mechanism of plant metabolites is not studied in depth, mainly focusing on the exploration of phenotypes, the research focuses on *in vitro* and *in vivo* studies in animals and lacks a large amount of data from clinical studies, plant metabolites may act on multiple targets and thus play an ameliorative and therapeutic role, so what is the most important mechanism needs to be investigated in-depth.

## 4 Common strategies for regulating intestinal flora to treat and prevent adipose metabolic diseases

Based on recent research and experimental findings, we have identified a number of common clinical approaches to ameliorate metabolic disorders by modulating gut microbes, including probiotic as well as antibiotic use, dietary fiber intake and Fecal microbiota transplantation (FMT), which we have summarized and generalized.

### 4.1 Probiotics and prebiotics

Probiotics and prebiotics regulating the intestinal flora is an emerging and promising therapeutic approach widely used in the prevention and treatment of NAFLD, mainly due to the fact that probiotics and prebiotics administration can repair the damaged intestinal barrier and thus restore its function (Fukui, 2015). This role is mainly based on its antimicrobial activity, which is effective against pathogens and reduces the number of pathogenic microorganisms (Paolella et al., 2014). At the same time, prebiotics can promote the growth and activity of probiotics, activating beneficial bacteria (Duarte et al., 2017). The regulation of probiotics and prebiotics restores the intestinal flora to a healthy balance. Bergheim et al. (2008) demonstrated in a 2008 study that neomycin and polymyxin B significantly reduced hepatic lipid deposition by reducing endotoxin translocation. In addition, another preclinical study found that antibiotics can modulate portal bile acid levels by inhibiting intestinal bacteria, thereby reducing liver inflammation (Janssen et al., 2017). Gut flora is a complex ecosystem that is influenced by many factors such as diet, environment and genetics. The intake of probiotics and prebiotics may be limited by the intestinal environment, thus limiting their effectiveness.

### 4.2 Nutrients and dietary components

Dietary fiber intake is a favorable factor in resistance to NAFLD progression (Xia et al., 2020). Fiber is degraded to short-chain fatty acids through fermentation by intestinal flora (Tan et al., 2014) involved in inflammation-related physiological processes. Increased nutritional fiber intake improves hepatic steatosis and liver function, while enhancing hepatic barrier function and decreasing intestinal permeability (Krawczyk et al., 2018). Wang Yong’s research team published a paper in the Journal of Functional Foods demonstrated that the combination of ferulic acid with arabinoxylan or β-glucan significantly improved glucose tolerance and maintained intestinal homeostasis in mice fed a high-fat diet (Fang et al., 2024). In contrast, chitosan COST was shown to improve hepatic lipid metabolism in HFD-induced NAFLD mice by modulating the expression of lipotoxicity-induced related inflammatory factors in the gut microbiota (Zhang et al., 2023). Although there is some research suggesting that nutrients and dietary fiber may be beneficial in ALD and NAFLD, there is still a lack of long-term, large-scale clinical trials to validate their effects. Therefore, it is unclear whether long-term intake of these nutrients is an effective treatment for these liver diseases.

### 4.3 Fecal microbiota transplantation

Fecal microbiota transplantation (FMT) is an emerging therapeutic approach to transfer fecal microbiota and metabolites from healthy donors to patients who need to rebuild their gut microbiota (Zhao et al., 2023). Clinical data demonstrated that FMT therapy effectively attenuated high-fat diet-induced steatohepatitis, resulting in a significant reduction in intrahepatic lipid accumulation and decreased intrahepatic expression of pro-inflammatory cytokines (e.g., IFN-γand IL-17) (Aron-Wisnewsky et al., 2020b), as well as restoring intestinal diversity, increasing the number of anaplastic bacilli, and decreasing the number of actinomycetes and thickened walled bacilli (Aron-Wisnewsky et al., 2020a). Ferrere et al. (2017) performed FMT on alcohol-resistant donor mice (alcohol-fed mice without alcohol-induced liver lesions) to alcohol-sensitive recipient mice (alcohol-fed mice with liver lesions) and found that FMT protected alcohol-sensitive mice from alcohol-induced consumption of Bacteroidetes mimosus. FMT treatment can involve two major drawbacks: safety and uncertainty of efficacy. FMT transfers fecal suspension from a healthy donor to the patient’s intestine and therefore carries the risk of transmitting disease, infection and allergic reactions. In addition, it may lead to infection in the patient because the fecal sample may contain undesirable microorganisms. Although there are some preliminary studies suggesting that FMT may positively affect liver function in patients with certain liver diseases, its exact therapeutic effect is unknown. There is a lack of large-scale, randomized controlled clinical trials to validate the effectiveness of FMT in ALD and NAFLD.

### 4.4 Combination of natural products with other biological agents

The combination of natural products and other biological agents for the treatment of NAFLD can give full play to greater advantages. On the basis of the pharmacological active effect on natural products, the combination of other biological agents synthesised lipid-lowering drugs, liver-protecting drugs and other drugs can effectively improve the clinical symptoms and biochemical indexes of patients (Gao et al., 2016). Microecological agents such as probiotics and prebiotics are used to regulate the balance of intestinal microorganisms by supplementing probiotics, prebiotics and other microecological agents, which can further consolidate the therapeutic effect and prevent disease recurrence. For example, Li and Gu (2022) investigated the combined effects of bicyclol and BBR on Western diet (WD)-induced steatosis and WD/CCl4-induced NASH in mice. Combination of natural products with other compounds is an effective way to improve NAFLD (Andreasen et al., 2023). Combined theories and methods to rebuild intestinal microecology and improve insulin resistance and fat metabolism are an effective way to improve NAFLD (Cui et al., 2023).

**FIGURE 2:**
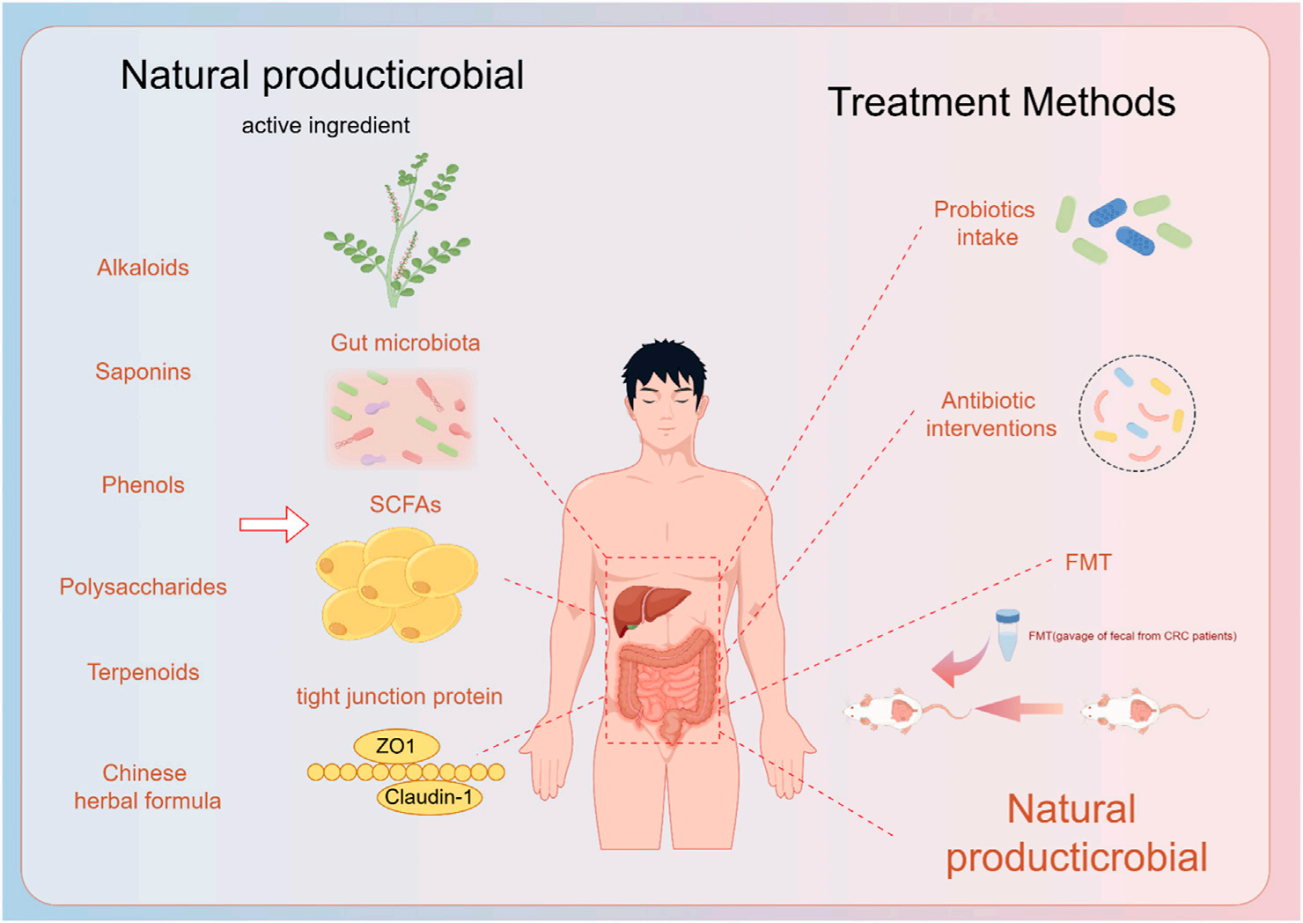
Natural products and their extracts play a role in the prevention and treatment of fatty liver through the gut-liver axis, and now the treatment of the gut-liver axis.

Since NAFLD was described in the 1980s, a large number of experimental studies and clinical trials have been conducted in China to investigate the efficacy of TCM in the treatment of NAFLD. Herbal medicines for the treatment of diseases are safe for clinical use and as of now dance adverse effects (Zhang et al., 2020; Yang et al., 2021). Consider the long-term use of botanical drugs and Western medicines, which has a broader perspective for rebuilding the intestinal microecology and improving NAFLD (Figure 2).

## 5 Discussion and prospect

The gut-liver axis plays a crucial role in the pathogenesis of NAFLD/ALD. Dysbiosis, increased gut permeability and gut-derived endotoxins are closely related to NAFLD/ALD development. Researchers have found dysregulation in the host-microbe interaction in various liver disease models, with impaired gut barrier exacerbating liver inflammation and disease progression. Clinical evidence demonstrates alterations in gut microbiota composition and metabolic products in NAFLD and ALD patients, affecting liver metabolic signaling pathways. Current treatments for NAFLD/ALD primarily involve probiotics, prebiotics, dietary fiber intake and FMT, but their limitations have shifted researchers’ focus toward natural products. Natural products can avoid the adverse effects of probiotics prebiotics and dietary fiber intake treatment due to individual differences. There are also some individual rejection as well as safety-type issues with probiotic prebiotics and FMT treatments, which contribute to the lack of effective treatment for NAFLD/ALD. The current researchers’ exploration of natural products for the treatment of NAFLD/ALD by regulating the enterohepatic axis mainly includes the directions of adjusting the intestinal flora, improving the intestinal mucosal barrier function, and reducing the release of endotoxin.

However, Our in-depth investigation reveals that the mechanism of NAFLD/ALD treatment by natural products through the intestinal flora is less studied at the histological level, such as only using 16S rDNA high-throughput sequencing of the intestinal flora or macro-genomic sequencing of the intestinal flora to characterize the difference in the flora, and lacks the analysis of functional metabolism molecules that are further associated with it, as well as the excavation and validation of the downstream mechanism of action. How natural products modify the composition and structure of the intestinal flora to regulate the production of intestinal metabolites, intervene in inflammation development and influence the processes of lipid metabolism in the body remains unclear. Natural products usually contain multiple active ingredients, which also gives them multiple mechanisms of action and targets of action that can affect the pathologic process of NAFLD/ALD in multiple ways simultaneously. Collating the current research we found that the mechanism of natural products exerting NAFLD/ALD effects by influencing immune cell function is becoming clearer, while the relationship between immune cells and intestinal flora is very close. On the one hand, gut flora disorders can lead to abnormal activation of the immune system, exacerbating liver inflammation and the development of NAFLD/ALD, on the other hand, an abnormal immune system response can further interfere with the balance of gut flora, creating a vicious cycle. Therefore, the interaction between intestinal flora and immunity plays a key regulatory role in the occurrence and development of NAFLD/ALD, should also be the focus and direction of research on the mechanism of natural product treatment of NAFLD/ALD through the enterohepatic axis.

In summary, we found that exploring the interaction mechanism between natural products and intestinal flora is expected to bring new breakthroughs in the treatment of fat-related metabolic diseases, as well as new opportunities for the application of natural products to improve the disorders of glucose and lipid metabolism and related diseases.
